# Domains of scale in cumulative effects of energy sector development on boreal birds

**DOI:** 10.1007/s10980-023-01779-8

**Published:** 2023-10-25

**Authors:** Andrew D. Crosby, Lionel Leston, Erin M. Bayne, Péter Sólymos, C. Lisa Mahon, Judith D. Toms, Teegan D. S. Docherty, Samantha J. Song

**Affiliations:** 1 Department of Biological Sciences, University of Alberta, Edmonton, AB Canada; 2 Alberta Biodiversity Monitoring Institute, University of Alberta, Edmonton, AB Canada; 3 Environment and Climate Change Canada, Whitehorse, YT Canada; 4 Environment and Climate Change Canada, Edmonton, AB Canada

**Keywords:** Anthropogenic disturbance, Biodiversity, Boreal conservation, Oil and gas footprint, Land use change, Scale dependence

## Abstract

**Context:**

Industrial development in Canada’s boreal forest creates cumulative environmental effects on biodiversity. Some effects may be scale-dependent, creating uncertainty in understanding and hindering effective management.

**Objectives:**

We estimated cumulative effects of energy sector development on distributions of sixteen migratory songbird species at multiple spatial scales within the boreal region of Alberta, Canada, and evaluated evidence for scale domains in species responses.

**Methods:**

We used a hierarchical, multi-scale sampling and modelling framework to compare effects of oil and gas footprint on songbirds at five spatial scales. We used Bayesian Lasso to facilitate direct comparison of parameter estimates across scales, and tested for differences in grouped parameter estimates among species.

**Results:**

We found consistent scale-dependent patterns across species, showing variable responses to development occurring at the smallest scale, little effect at intermediate scales, and stronger, mainly positive effects at the largest scales. Differences in grouped parameter estimates across scales showed strong evidence for scale domains in the response of songbirds to energy sector development.

**Conclusions:**

We concluded that variable effects at the smallest scale represented individual habitat selection, while larger scale positive effects reflected expanding distributions of open habitat- and disturbance-associated species in areas of high oil and gas footprint. Our results show that single-scale analyses do not reflect population processes occurring at other scales. Future research on linking patterns at different scales is required to fully understand cumulative effects of land use change on wildlife populations.

**Supplementary Information:**

The online version contains supplementary material available at 10.1007/s10980-023-01779-8.

## Introduction

Many forested areas of the globe are undergoing increased industrial development, leading to changes in biodiversity and degradation of ecosystem services (Watson et al. 2018). Development can affect ecosystems through the disturbances they create individually and collectively. Natural resource development creates many small-scale disturbances, with localized impacts on ecosystem composition and structure that have been reasonably well studied (e.g. Pickell et al. 2015; Mahon et al. 2019). At larger scales, individual disturbance types can interact across space and time, resulting in a greater combined effect on ecosystem processes known as cumulative effects (Venier et al. 2021). Currently, our understanding of cumulative effects is far less clear than our basic knowledge about how individual disturbances influence biodiversity (Venier et al. 2021).

In the boreal region of western North America, expansion of energy development is creating novel landscape patterns well outside the historic range of variability (Pickell et al. 2015). These changes affect the amount and distribution of wildlife habitat, and can have profound effects on biodiversity (Venier et al. 2014). Effective regulation of development requires understanding how terrestrial species respond to anthropogenic disturbance by the energy sector (hereafter, “footprint”) (Mahon & Pelech 2021). Footprint types can differ greatly in intensity and spatial extent (Mahon et al. 2019). For example, energy production facilities have a large impact in the immediate area (e.g. complete removal of vegetation, noise, pollution), but are limited to a relatively small proportion of the landscape when viewed at larger spatial extents. Seismic lines, on the other hand, are narrow linear features that have relatively low impact in the immediate area (used for a short time, then left to regenerate or actively restored), but are ubiquitous and create vast amounts of edge habitat throughout the western boreal forest. Roads and pipelines are less common but more frequently disturbed than seismic lines, resulting in a greater immediate impact on vegetation, and tend to have local impacts intermediate between industrial facilities and seismic lines (Pickell et al. 2015). Individual wells are small (typically < 1 ha), produce less edge than seismic lines, and can be left to regenerate natural vegetation after well abandonment. However, wells also cover a total area similar to seismic lines, so their cumulative area and impact is also large. Thus, there are two aspects of cumulative effects that must be considered: the combination of footprint types, and the accumulated area of footprint across the landscape. Studies conducted at single, fixed scales have found evidence for disturbance-specific and cumulative effects of footprint on biodiversity (e.g. Bayne et al. 2016; Fisher & Burton 2018; Mahon et al. 2019; Leston et al. 2023). However, little is known about how patterns of cumulative footprint influence species at different spatial scales.

In ecology, spatial scale of sampling affects conclusions about species’ response to environmental conditions, and choice of appropriate scale is critical to answering ecological and management questions (Wiens 1989). Different processes may become dominant at different scales, sometimes leading to contradictory conclusions among studies of the same phenomena (Turner et al. 1989; Wiens 1989). Wiens (1989) formulated the concept of ‘domains of scale’, suggesting there may be regions of the scale spectrum over which ecological relationships remain consistent, separated by transition zones where relationships are highly variable and unpredictable. Bestelmeyer et al. (2003) linked scale domains to specific ecological processes governing species diversity, proposing habitat, landscape, and geographic domains corresponding to individual home range selection, landscape scale distribution, and geographic range, respectively. Investigating the potential role of these domains is critical to understanding how individuals and populations respond to changing environmental conditions, and thus how footprint affects wildlife populations and biodiversity.

The issue of scale and how it influences management decisions for wildlife conservation is particularly important for birds in the western boreal forest of North America. The importance of the boreal region for sustaining North American bird populations is well established, to the point where it is commonly referred to as North America’s bird nursery (Blancher & Wells 2005). Many boreal bird species associated with mature forests respond negatively to energy development at local scales, while habitat generalists and species associated with younger forests and open habitat types tend to be positively affected (Bayne et al. 2016; Mahon et al. 2019; Leston et al. 2023). These effects can lead to changes in bird community composition and reductions in diversity, thus increasing the rate of biotic homogenization and consequent loss of ecological functions (Olden 2006; Mahon et al. 2016). Quantifying scale-dependent effects of anthropogenic disturbance on bird populations is an important step in understanding consequences to the ecosystem (Mahon et al. 2016).

**Fig. 1:**
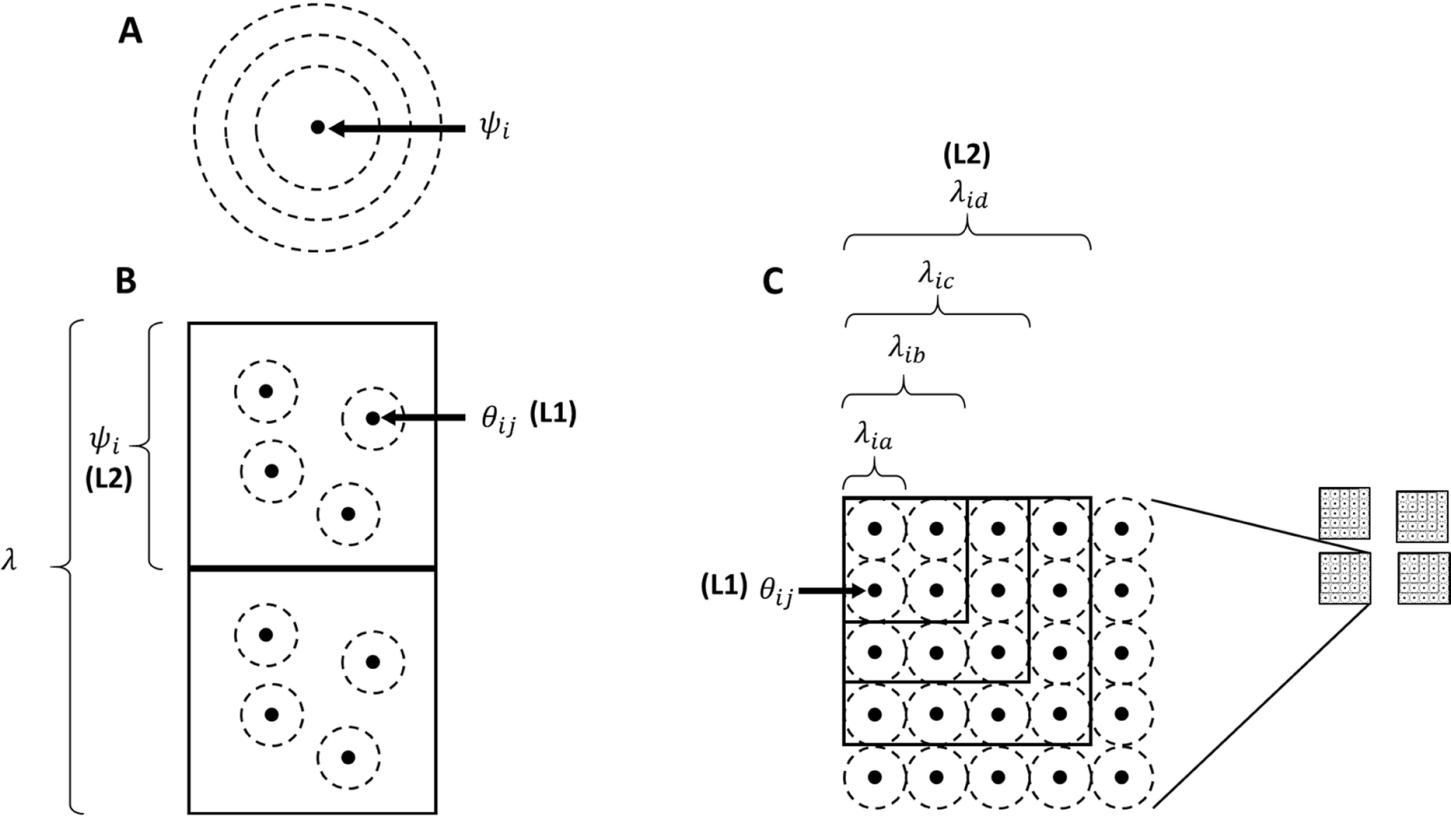
Three types of multiscale occupancy sampling designs, where $\psi$ refers to sample unit occupancy probability, $\theta$ refers to subsample occupancy probability, and $\lambda$ refers to expected number of occupied sites or proportion of area occupied. Bold captions in parentheses refer to the organizational level of the study design: **A** multiple radii around a sampling point, where inference is being made at the point (grain) and study area (extent) scales; **B** multi-level hierarchical where points are subsamples within blocks (**L1**), blocks are samples within the study area extent (**L2**), and subsample grain and sample block extent remain constant; and **C** multi-level, multi-scale hierarchical designs where subsample grain (**L1**) remains constant, and sample block extent (**L2**) changes

In an ecological modeling framework, there are different ways to characterize scale-dependent effects of environmental variables (McGarigal et al. 2016). Many studies have tested effects of landscape variables at multiple scales around individual sampling points (multi-scale design, Fig. 1A, McGarigal et al. 2016). For example, Bayne et al. (2016) investigated how detection of bird responses to disturbance changed with sampling radius. Less-often, researchers have modeled species occupancy or abundance hierarchically by defining spatially nested subsets of observations (multi-level design, McGarigal et al. 2016), typically using different sets of variables at each level of the hierarchy (Fig. 1B, sensu Mordecai et al. 2011). In this multi-level design, the sampling extent at each level typically stays fixed, either at specific dimensions (e.g. block size) or ecological level (e.g. watershed), and the metric of interest is occupancy or abundance at each level. However, if the goal is to understand the cumulative effects of disturbances across large spatial extents, it is also important to understand how landscape extent influences model results and conclusions. Such inference requires a multi-level, multi-scale sampling design (e.g. Fig. 1C), where sampling extent at the second level or greater can also vary.

Here, we employ a multi-level, multi-scale sampling design and modeling framework to quantify cumulative effects of oil and gas footprint on boreal songbird populations at different spatial scales in the western boreal region of Canada. Our objectives were to compare bird species responses to footprint among scales, and examine evidence for domains of scale in bird response to energy sector footprint. We considered three response types: **A**) a simple, direct effect of loss or alteration of native vegetation, regardless of footprint type (total area of habitat disturbed); **B**) independent direct effects among footprint types (additive effects); and **C**) interactive effects of different footprint types. We hypothesized that, if there are domains of scale in songbird responses to footprint, we would see consistent scale-dependent patterns in responses among species. Although our application is specific to birds in the western boreal forest region of Canada, our unique sampling design and modeling framework is applicable to other regions and taxa.

## Methods

### Study area

**Fig. 2:**
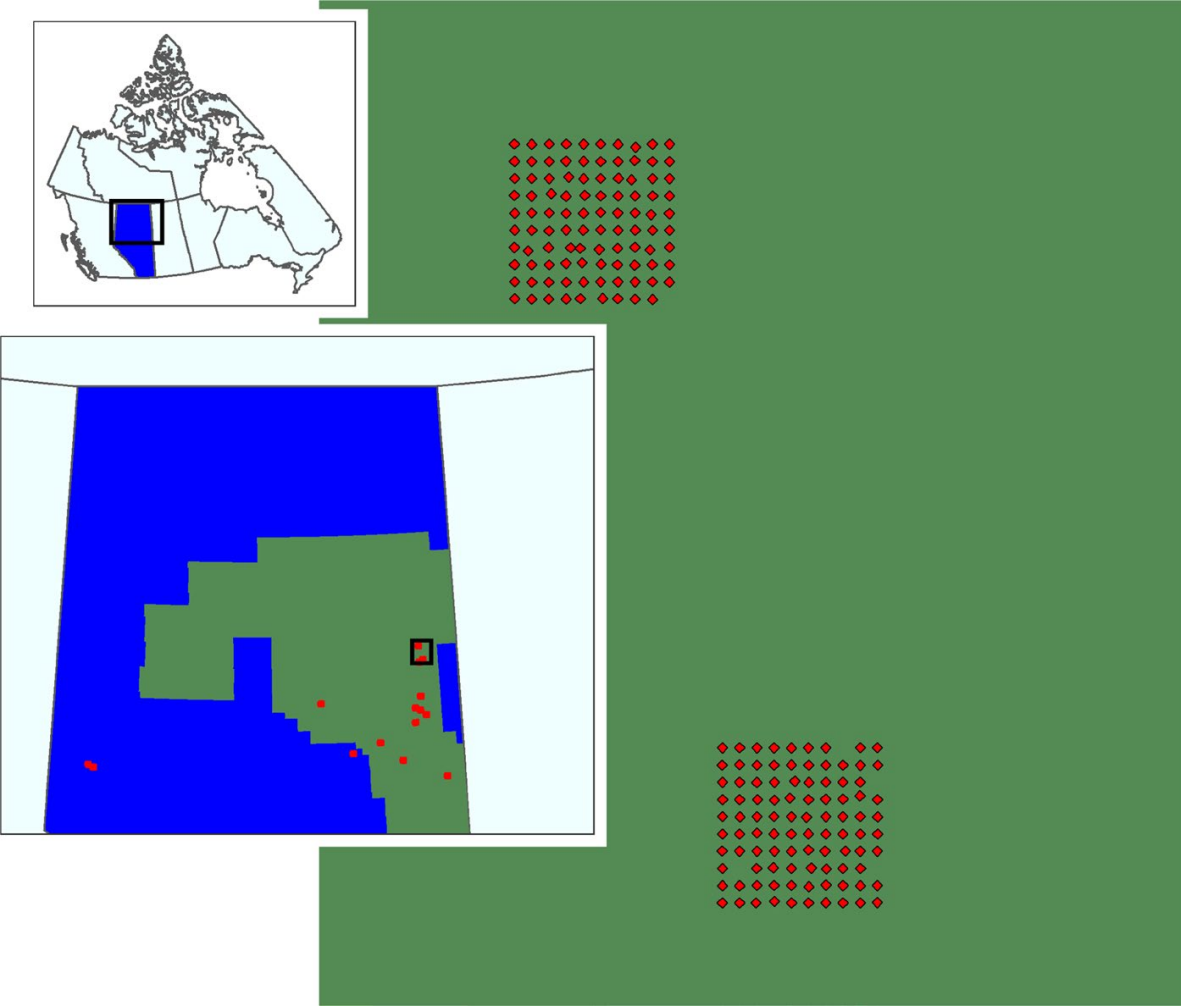
Study area showing the location of the Oil Sands Region (green) in Alberta (blue), and the locations and arrangement of big grid sampling points (red)

Our study area was the boreal forest region of northern Alberta, Canada, with most sampling locations occurring in the Mid-boreal Uplands ecoregion within the Athabasca Oil Sands region, and a small subset in the Boreal Transition, Western Alberta Upland, and Western Boreal ecoregions (Fig. 2, Ecological Stratification Working Group 1995). Throughout the region, dominant upland trees include trembling aspen (*Populus tremuloides*), balsam poplar (*Populus balsamifera*), white spruce (*Picea glauca*), and jack pine (*Pinus banksiana*). Black spruce (*Picea mariana*) and tamarack (*Larix lariciana*) occur in wet lowlands along with bogs, fens, and marshlands. Energy sector activities in the study area frequently create disturbances such as seismic lines, pipelines, well-pads, industrial plants, power transmission lines, and roads.

### Sampling design and data collection

We used data on bird species occurrence, habitat, and energy sector (oil and gas) footprint derived from a large-scale, grid-based sampling design (hereafter “big grids”). Each big grid consisted of a 10 × 10 grid of points spaced 600 m apart (Fig. 2), designed to assess ecological impacts of Steam Assisted Gravity Drainage (SAGD) facilities in Alberta’s Oil Sands region. Each SAGD site (hereafter “high footprint”) contains a central processing facility where extracted bitumen is pumped through pipelines from injection and extraction wells. The SAGD lease area around the facility (typically 50–100km^2^) contains unimproved roads, seismic lines, and exploration wells, and disturbance levels are higher than the surrounding landscape. Based on vegetation conditions at high footprint sites, we chose two additional grids: intermediate disturbance, and low disturbance. Intermediate disturbance grids were in areas where considerable energy exploration had taken place but no SAGD extraction had occurred. Low footprint grids were placed in areas of the study region where parks prevented bitumen development or a low probability of future energy development was expected. Our goal was to get a similar number of grids in each footprint category in landscapes dominated by upland vs lowland forest. We had a total of 15 grids for our study: 5 in highly disturbed landscapes (with SAGD facilities); 4 in intermediate landscapes (exploration but no SAGD extraction); and 6 in low disturbance landscapes.

To collect data on bird species occurrence, autonomous recording units (ARU) were placed at each grid point (SM2, SM3, SM4 + by Wildlife Acoustics Ltd.). All sampling from a given grid was done in the same year, in 2014–2019. Each point was sampled for $\ge$ four days. Technicians listened to recordings from each point, and noted all individually distinguishable birds and the time at which the individual was first heard. Recordings were filtered to remove those with excessive noise, and by selecting only those occurring between May 19 and July 11, and between 04:00 and 10:00 h, as these are the main periods of breeding and singing activity for birds in northern Alberta (Charchuk & Bayne 2018). Most transcribed recordings were recorded near dawn (04:00–06:00). Although we tried to select four surveys from different days, if poor weather conditions limited choice, we selected additional times from the same day. Our final dataset consisted of detection/non-detection data for all bird species heard within the first three minutes of a recording, at all points where $\ge$ two recordings were available from the same year.

We summarized vegetation and footprint at each point using data from the Alberta Biodiversity Monitoring Institute (ABMI; https://abmi.ca/home.html). ABMI derives vectorized natural land cover classes and forest age from the Alberta Vegetation Inventory (AVI), which uses interpretation of medium-scale (1:60,000 or 1:40,000) aerial photographs updated with forest harvest and wildfire data. Year-specific, vectorized footprint layers are created from a combination of AVI and SPOT 6 satellite imagery interpretation, and are combined with vegetation types to create wall-to-wall vegetation and footprint maps for the province. For more detailed information on vegetation and footprint data products used in our analysis, see Alberta Biodiversity Monitoring Institute (2017) and Alberta Biodiversity Monitoring Institute (2019), respectively, or contact ABMI directly at abmiinfo@ualberta.ca.

Our data consisted of area covered by each of seven upland and lowland forest types, six non-forested wetland types, four non-forested upland types, and twenty-seven footprint types (Appendix A; Tables A1 and A2) within a 150 m circular buffer (7 ha) surrounding each point and a 600 m x 600 m (36 ha) cell centered on each point. Forest types were further separated into ten age classes (0–9, 10–19, 20–39, 40–59, 60–79, 80–99, 100–119, 120–139, 140–159, and 160 + years old). In the original vegetation data, upland forest stands (Deciduous, Mixedwood, Pine, and White Spruce) harvested within the past 59 years were designated as distinct from unharvested stands, and were separated into age classes of 0–9, 10–19, 20–39, and 40–59. (Appendix A; Table A1). Because our study was focused on understanding effects of oil and gas footprint, we accounted for harvest through harvest’s effect on forest age only, and did not consider harvested stands to be a separate category from unharvested stands in our analysis. We aggregated vegetation data into proportion of area covered by each of 6 forest types in each of the ten age classes, non-forested uplands, and non-forested wetlands (hereafter, habitat types) within the 150 m radius of each point (Appendix A; Table A1). We aggregated footprint into proportion of area covered by four classes: seismic lines; wide linear disturbances (e.g. roads, power lines, pipelines); well sites; and industrial in the 600m^2^ grid cell centered on each point. [Appendix A; Table A2, sensu Mahon et al. (2019)]. We summarized footprint at larger spatial extents with a moving window over the grids at sizes of 2 × 2, 3 × 3, 4 × 4, and 5 × 5 grid cells, where each 36 ha grid cell contained a single sampling point. The moving windows yielded 1,215, 960, 735, and 540 blocks available for sampling in the 2 × 2, 3 × 3, 4 × 4, and 5 × 5 window sizes, respectively. To generate equal sample sizes among scales, we randomly selected 300 samples at each extent, including the 1 × 1 extent. For our purposes, point-level refers to a grid point where birds were sampled, and the 150 m radius around the point from which habitat variables were derived, and block-level refers to points (and cells) aggregated within the different window sizes, where there are five block sizes (1 × 1–5 × 5), and a 1 × 1 block is a 36 ha cell containing a single grid point.

**Table 1:** Probability of a sample containing each type of oil and gas footprint, and median and interquartile ranges of area (ha) within sample units, across spatial scales

Scale	Footprint type				
	Seismic	Wide linear	Wells	Industry	Any footprint
1 × 1	0.858	0.658	0.545	0.241	0.948
(36 ha)	0.42 (0.134, 0.972)	0.229 (0, 2.11)	0.278 (0, 1.142)	0 (0, 0)	2.044 (0.634, 5.012)
2 × 2	0.98	0.875	0.717	0.405	0.998
(144 ha)	0.906 (0.436, 1.825)	1.494 (0.182, 4.154)	0.83 (0, 2.372)	0 (0, 0.958)	4.898 (1.567, 10.242)
3 × 3	0.999	0.948	0.797	0.541	1
(324 ha)	1.451 (0.804, 2.827)	3.264 (0.564, 6.353)	1.451 (0.267, 4.169)	0.116 (0, 2.231)	8.648 (2.549, 16.423)
4 × 4	1	0.978	0.849	0.641	1
(576 ha)	2.024 (1.22, 3.605)	4.724 (1.198, 8.193)	2.065 (0.327, 5.865)	0.4 (0, 4.533)	11.451 (3.995, 26.369)
5 × 5	1	0.993	0.885	0.696	1
(900 ha)	2.593 (1.545, 4.539)	6.013 (2.008, 10.274)	2.609 (0.325, 8.795)	0.617 (0, 5.855)	14.829 (5.85, 35.196)

Each scale represents a square grid of 36 ha cells. The column 'Any footprint' refers to the probability that ≥ 1 of the 4 footprint types occurs in the cell

**Table 2:** Scale-dependent best models based on pseudo Bayesian Model Averaging weights (in parentheses), and strongest explanatory variables (below), for response of sixteen passerine species to oil and gas footprint in Alberta, Canada, sorted by habitat association

Species	Habitat	Scale				
		1 × 1	2 × 2	3 × 3	4 × 4	5 × 5
Swainson's Thrush	CO	Total (0.7)	Additive (0.95)	Interactive (0.96)	Interactive (0.99)	Total (1)
		T(−)				T( +)
Tennessee Warbler	CO	Additive (0.35)	Additive (0.7)	Total (1)	Interactive (0.96)	Interactive (0.93)
				T( +)		I( +)
Blue-headed Vireo	CO	Total (0.39)	Additive (0.84)	Interactive (1)	Total (1)	Additive (1)
		T(−)				
Ovenbird	DE	Total (0.39)	Total (0.87)	Interactive (0.7)	Interactive (1)	Total (1)
			T( +)			T( +)
Mourning Warbler	DE	Interactive (0.36)	Additive (0.73)	Total (0.71)	Total (1)	Total (1)
			W( +), I( +)		T( +)	T(+ +)
Rose-breasted Grosbeak	DE	Interactive (0.36)	Additive (0.4)	Interactive (0.97)	Total (0.54)	Total (1)
					T(−)	T(−)
Connecticut Warbler	DE	Total (0.88)	Interactive (0.68)	Additive (1)	Total (1)	Additive (0.45)
		T(−)	WL(−)			
Yellow-rumped Warbler	GE	Interactive (0.35)	Total (0.37)	Total (0.85)	Total (0.78)	Additive (0.92)
Magnolia Warbler	GE	Additive (0.35)	Interactive (0.52)	Interactive (0.57)	Additive (1)	Total (1)
			WL(−), I( +)	W( +), I( +)	W( +), I( +)	T(+ +)
Lincoln's Sparrow	OP	Additive (0.53)	Interactive (1)	Interactive (1)	Total (1)	Total (1)
		WL( +), I( +)				T( +)
Alder Flycatcher	OP	Total (0.66)	Total (0.59)	Interactive (1)	Interactive (1)	Total (1)
		T(+ +)			I( +)	T( +)
Clay-colored Sparrow	OP	Additive (0.5)	Total (0.81)	Additive (1)	Interactive (0.52)	Additive (1)
		WL( +), W( +), I(+ +)			I( +)	I( +)
Chipping Sparrow	SB	Interactive (0.36)	Total (0.53)	Additive (0.64)	Total (1)	Total (1)
			T( +)		T(+ +)	T(+ +)
Dark-eyed Junco	SB	Interactive (0.37)	Interactive (0.48)	Additive (0.9)	Additive (0.98)	Total (0.99)
Ruby-crowned Kinglet	SB	Additive (0.45)	Additive (0.53)	Interactive (1)	Total (1)	Additive (0.98)
					T(+ +)	
Palm Warbler	SB	Additive (0.36)	Interactive (0.51)	Additive (1)	Additive (1)	Total (1)
						T(−)

Under 'Habitat': *CO* upland conifer, *DE* deciduous, *GE* generalist, *OP* open lands, *BS* black spruce. For explanatory variables: *S* seismic lines, *WL* wide linear, *W* wells, *I* industrial, and *T* total. A ' + ' or '−' following the variable indicates weak support (single) or strong support (double) for a positive or negative relationship, respectively. No variables associated with a model indicates none of the variables had a 20% Bayesian Credible Interval (CI) that did not include 0. Strong support indicates the 95% CI for that variable did not include 0

### Multi-level hierarchical occupancy model

**Fig. 3:**
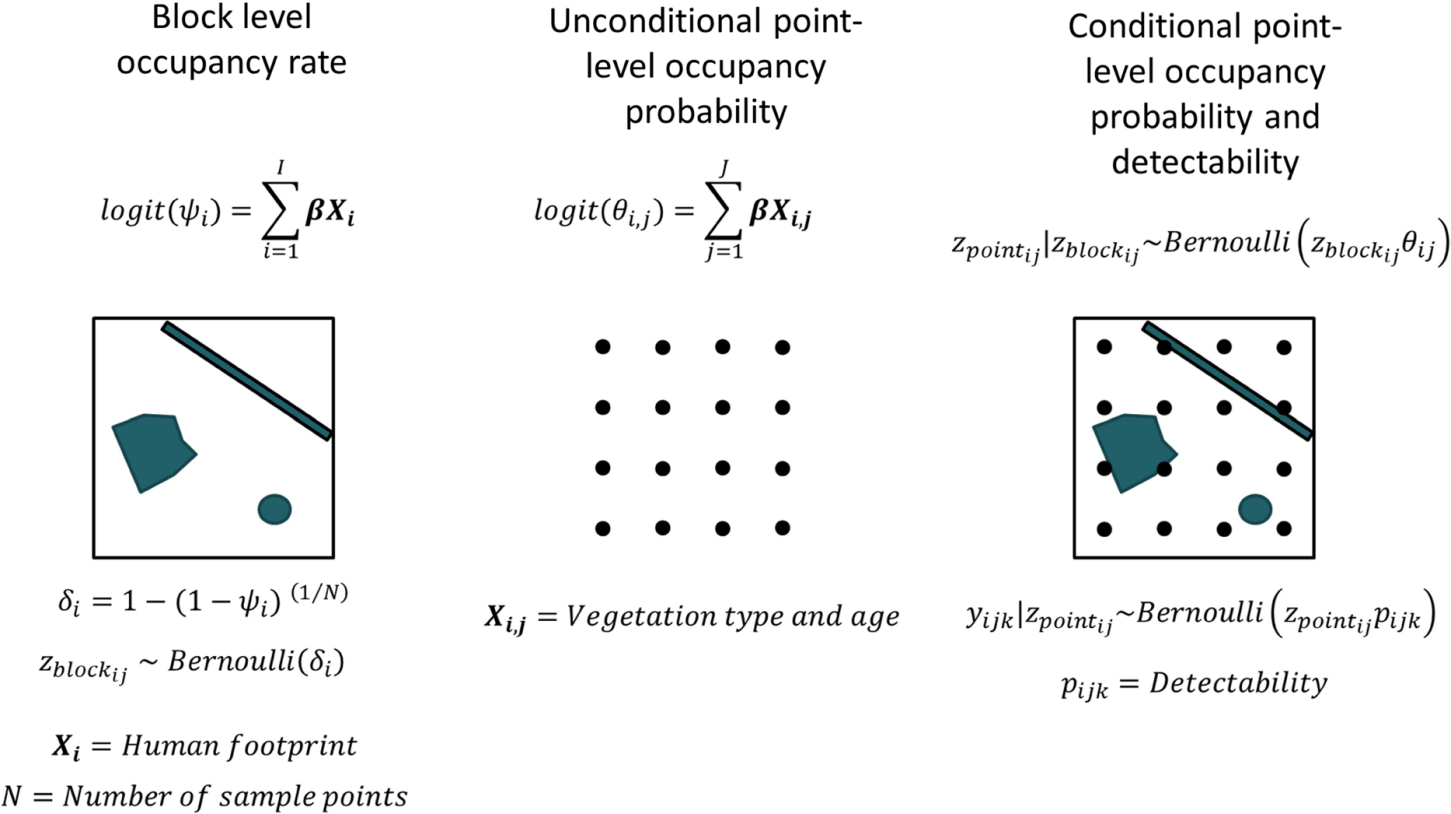
Hierarchical occupancy model diagram, showing the estimation of: block-level occupancy rate (${\delta }_{i}$) in block *i* as a function of oil and gas footprint (blue polygons); unconditional point-level occupancy probability (${\theta }_{i,j}$) at point $i,j$ as a function of dominant vegetation and forest age; conditional point-level occupancy (${z}_{poin{t}_{i,j}}$) as a joint probability of block-level occupancy rate and point level occupancy probability; and detection (${y}_{i,j,k}$) at survey *k* as a function of occupancy status and (${z}_{poin{t}_{i,j}}$) and detectability (${p}_{i,j,k}$)

We modeled the relationship between bird species occupancy and footprint using a hierarchically structured occupancy model that explicitly separated block-level occupancy rate, as a function of footprint, from point-level occupancy probability within the block as a function of dominant forest type and forest age (Fig. 3). Our model conditioned point-level occupancy probability on occupancy rate at the block level (the probability that a given point within the block is occupied), rather than occupancy probability (the probability that the species occurs within the block), to control for the effect of block size on occupancy probability, which allowed us to directly compare models across scales. Thus, at the block level, we calculated occupancy rate $\delta$ in block $i$ as the probability that the species occurred at any given point sampled within the block, such that

$$logit\left({\psi }_{i}\right)=\sum {{\varvec{\upbeta}}}_{\mathbf{b}\mathbf{l}\mathbf{o}\mathbf{c}\mathbf{k}}{\mathbf{X}}_{\mathbf{i}},$$

and

$${\delta }_{i}=1-{\left(1-{\psi }_{i}\right)}^{1/{n}_{scale}},$$

where ${\psi }_{i}$ is the block-level occupancy probability, ${{\varvec{\upbeta}}}_{\mathbf{b}\mathbf{l}\mathbf{o}\mathbf{c}\mathbf{k}}{\mathbf{X}}_{\mathbf{i}}$ is the vector of block-level coefficients ${{\varvec{\upbeta}}}_{\mathbf{b}\mathbf{l}\mathbf{o}\mathbf{c}\mathbf{k}}$ and footprint covariates $\mathbf{X}$ in block $i$, respectively (including the intercept), and ${n}_{scale}$ is the number of sample points in each block at a given scale. Thus, the occupancy rate ${\delta }_{i}$ is derived from block-level occupancy probability, and is the variable upon which point-level occupancy probability is conditioned, such that for each point $j$ in block $i$, ${z}_{bloc{k}_{i,j}}\sim Bernoulli\left({\delta }_{i}\right)$, where ${z}_{bloc{k}_{i,j}}$ is the occupancy indicator (0 or 1) as a random realization of block-level occupancy rate (Fig. 3).

At the point level, we modeled unconditional occupancy probability as a function of dominant habitat type (the habitat type covering the most area within 150 m of the point) and mean area-weighted forest age within 150 m of the point, independent of block-level footprint (Fig. 3). Thus, point-level occupancy was estimated at a resolution of 7 ha. To reduce the influence of points in a mixture of habitats, or with large areas of footprint within the 150 m radius, we weighted observations according to the proportion of area covered by the dominant habitat type, where weight = 1 for proportions ≥ 0.75, linearly decreasing between 0.75 and 0.25, and 0 for < 0.25 proportions (Sólymos et al. 2020). Thus,

$$logit\left({\theta }_{ij}\right)=\sum {{\varvec{\upbeta}}}_{\mathbf{p}\mathbf{o}\mathbf{i}\mathbf{n}\mathbf{t}}{\mathbf{X}}_{\mathbf{i}\mathbf{j}},$$

where ${\theta }_{ij}$ is the unconditional point-level occupancy probability, ${{\varvec{\upbeta}}}_{\mathbf{p}\mathbf{o}\mathbf{i}\mathbf{n}\mathbf{t}}{\mathbf{X}}_{\mathbf{i}\mathbf{j}}$ is the vector of point-level coefficients ${{\varvec{\upbeta}}}_{\mathbf{p}\mathbf{o}\mathbf{i}\mathbf{n}\mathbf{t}}$ and habitat covariates $\mathbf{X}$ at point $j$ in block $i$. Consequently, ${z}_{poin{t}_{ij}}|{z}_{bloc{k}_{ij}}\sim Bernoulli\left({z}_{bloc{k}_{ij}}{\theta }_{ij}\right)$, where ${z}_{poin{t}_{ij}}$ is the conditional point-level occupancy indicator (0 or 1; Fig. 3).

We accounted for detection probability in our data using temporal subsamples at each point, such that the probability of detection $p$ at point $i,j$ during subsample $k$ was

$$logit\left({p}_{ijk}\right)=\sum_{k=1}^{K}{{\varvec{\upbeta}}}_{\mathbf{s}\mathbf{u}\mathbf{r}\mathbf{v}\mathbf{e}\mathbf{y}}{\mathbf{X}}_{\mathbf{i}\mathbf{j}\mathbf{k}},$$

where ${{\varvec{\upbeta}}}_{\mathbf{s}\mathbf{u}\mathbf{r}\mathbf{v}\mathbf{e}\mathbf{y}}$ is the vector of coefficients on detection probability, and ${\mathbf{X}}_{\mathbf{i}\mathbf{j}\mathbf{k}}$ is the vector of detectability covariates at point $i,j$ during subsample $k$. Thus, the observation data is ${y}_{i,j,k}|{z}_{poin{t}_{ij}}\sim Bernoulli\left({z}_{poin{t}_{ij}}{p}_{i,j,k}\right)$. We modeled detectability for all species as a function of Julian day and its quadratic, and time since sunrise.

### Comparison among scales

To meet our objective of comparing footprint effects on expected block-level occupancy rates among species and scales, we fit three separate models for each species at each scale: a model that used total footprint as the only predictor; a model using additive effects of individual footprint types (additive effects model); and a model with pairwise interactions of all footprint types (interactive effects model). Because we wanted to simultaneously compare model coefficients among scales and choose the best model at each scale, we employed a continuous model selection technique (Bayesian Lasso, sensu Gerber et al. 2015; Stevens & Conway 2019) that allowed us to identify a best model at each scale while still including all variables in the individual model types. Most studies utilizing model selection employ discrete selection, where a number of models with discrete sets of variables are compared. In discrete selection, the effect of a variable in a specific model is set to zero a priori by eliminating it from the model. Bayesian Lasso, by contrast, includes all variables of interest and forces coefficients of uninformative variables towards zero through the use of increasingly narrow prior distributions centered on zero (Hooten & Hobbs 2015). We implemented the Lasso procedure by using a Laplace prior (Gerber et al. 2015) on all non-intercept variables in the block-level portion of the model, specifying fifty candidate values of prior precision ranging from 0.1 (wide, uninformative priors) to 5 (essentially forcing all coefficients to near zero) on the log scale (Stevens & Conway 2019). We used Watanabe-Akaike Information Criterion (WAIC, Watanabe 2010) as our estimate of posterior predictive accuracy to determine optimal prior variance for each version of the model. To determine which model type (additive, interactive, or total) was best at each scale (i.e. highest posterior predictive accuracy), we compared models using pseudo Bayesian Model Averaging weights (pBMA, Höge et al. 2020). See Appendix B for a description and tutorial on running the models with the Bayesian Lasso implementation.

We fit models for 16 species of songbirds, choosing 2–4 representative species of 5 different habitat associations: species associated with upland coniferous forests, deciduous forests, open lands, or black spruce; and habitat generalists (sensu Mahon et al. 2019). The final set of species was chosen based on expert opinion from a list of 24 species that were detected at > 10% of sample plots, plus the blue-headed vireo, which was detected at just under 10% of the sample points. We chose this threshold to ensure there would be enough detections for fitting models. The species were chosen to represent the range of primary habitat associations within the region (Mahon et al. 2016). We fit models with the JAGS (Plummer 2003) and R (R Core Development Team 2017) programs using the jagsUI R package (Kellner 2015), with three chains of 50,000 iterations each, a burn-in of 40,000, and a thinning rate of 2. All non-footprint parameters, except for intercepts on block-level and point-level occupancy, were given uninformative priors, where ${\beta }_{i}\sim Normal\left(\mu =0,\tau =0.1\right)$*.* Intercepts for block-level and point-level occupancy were given vaguely informative priors, where ${\beta }_{1}\sim Normal\left(\mu =0,\tau =1\right)$*,* to facilitate model convergence. We used the loo package in R (Vehtari et al. 2020) to calculate WAIC scores and pBMA weights, and applied Bayesian bootstrap to the pBMA calculation.

We compared parameter estimates within each model type (total footprint, additive effects, and interactive effects) among species and across scales. Based on Bayesian Lasso results, we considered there to be no detectable effect if the 20% Bayesian Credible Interval (CRI) contained 0, weak evidence for an effect (i.e. high uncertainty) if the 95% CRI contained 0 but the 20% CRI did not, and strong evidence for an effect if the 95% CRI did not contain 0. These cutoff values acted as an index of the magnitude of effect represented by a variable.

To meet our second objective, we investigated evidence for domains of scale in two ways. As there is yet no formal, quantitative method to assess scale domains (Wheatley 2010), we first visually compared plots of scale-specific parameter estimates and model comparisons to look for consistent patterns among plots. Second, we compared parameter estimates for all independent parameters (additive and total models) and species among scales using the nonparametric Kruskal–Wallis test to look for evidence of overall differences among scales, and applied the pairwise Wilcoxon Rank Sum test with the Benjamini–Hochberg correction for multiple comparisons to test for pairwise differences in means between scales (sensu Wheatley 2010).

## Results

**Fig. 4:**
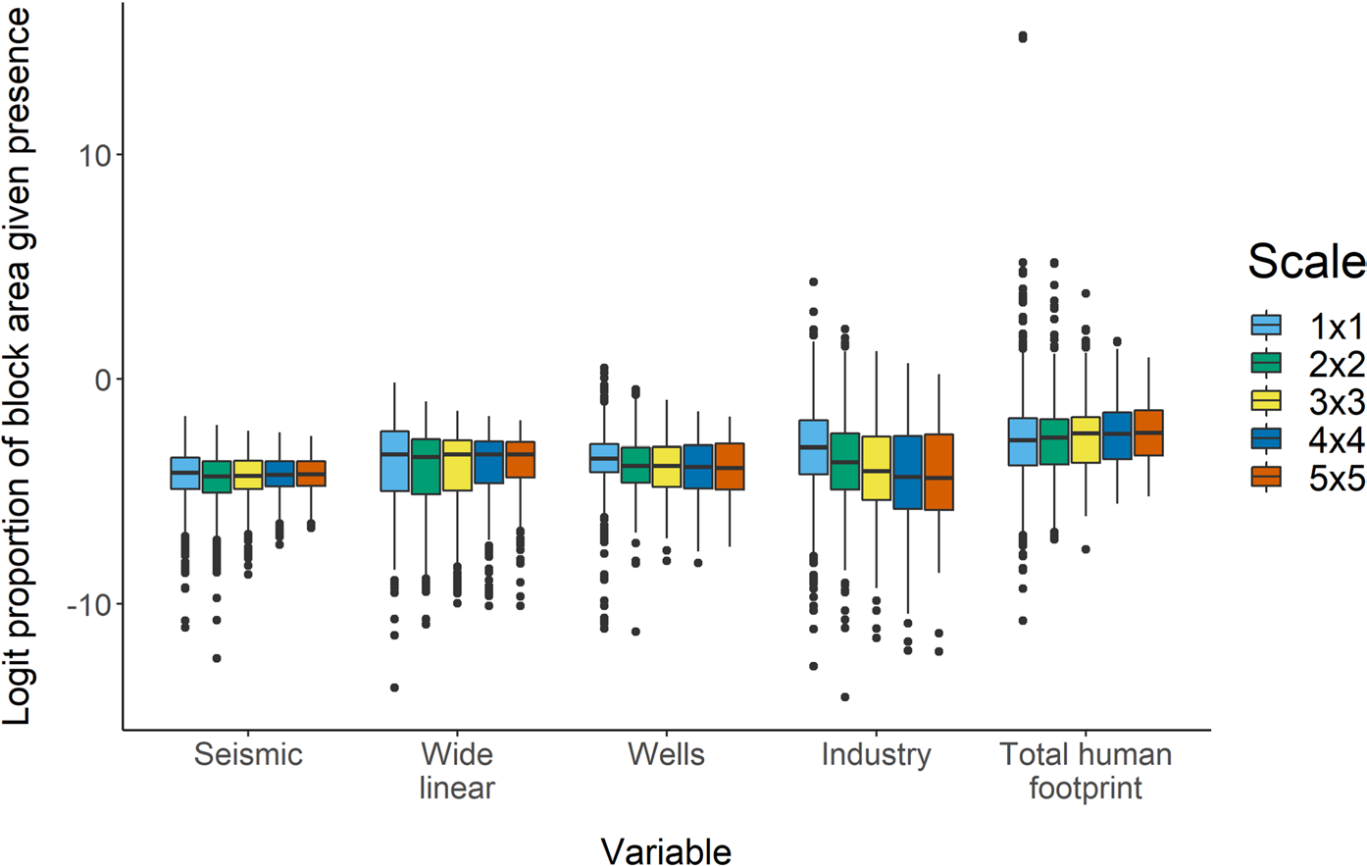
Logit proportion of area covered by individual oil and gas footprint types, conditional on the presence of that footprint type, and total footprint conditional on the presence of any footprint, at five spatial scales in the boreal forest region of Alberta, Canada. Scales represent square grids of points, where points are 600 m apart

We found the probability of a sample containing footprint was high, being 0.95 at the 1 × 1 scale and 1 for all other scales (Table 1). The most widely distributed footprint type was seismic lines, occurring in 98% of samples at the 2 × 2 scale and all samples at larger scales, while the industry category was the least widely distributed footprint type (Table 1). Median proportion of area in different footprint types, when present in a sample, was consistent across scales for seismic, wide linear, and wells, but decreased with increasing scale for industrial disturbance (Fig. 4). For seismic, wide linear and total HF, interquartile range and range of outliers decreased as scale increased, while for wells and industrial disturbance, interquartile range increased with increasing scale (Fig. 4).

**Fig. 5:**
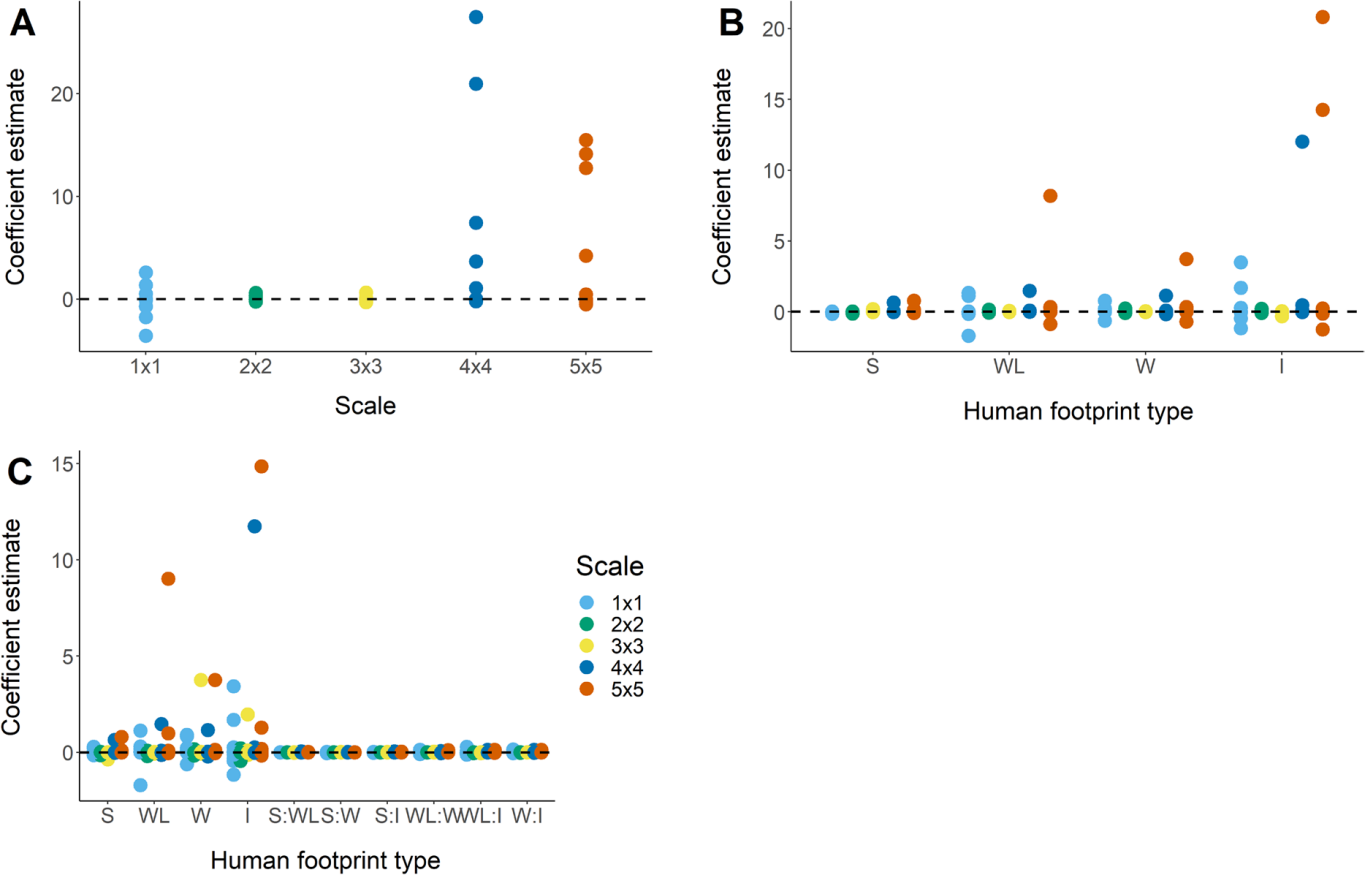
Coefficient estimates for effect of oil and gas footprint variables on occupancy rates among sixteen bird species at five spatial scales in the Boreal forest region of Alberta, Canada. Scales represent square grids of points, where points are 600 m apart. Plots represent: **A** total combined footprint effect; **B** independent effects of individual disturbance types; and **C** pairwise interactions between disturbance types. *S* seismic lines, *WL* wide linear, *W* well pads, *I* industrial development

There was a mix of positive and negative responses across species and scales to total footprint and individual footprint types (Fig. 5). The strongest negative effects of total footprint occurred at the 1 × 1 scale, while the strongest positive effects were at the 4 × 4 and 5 × 5 scales (Fig. 5A). In our additive effects models, seismic lines had the weakest effects, while industry had the strongest (Fig. 5B). All model types (total, additive, and interactive) showed the weakest effects at 2 × 2 and 3 × 3 scales (Figs. 5A–C). Industrial development had the strongest overall effect on species occupancy rates, especially at the 1 × 1 and 5 × 5 scales (Fig. 5B and 5C).

**Fig. 6:**
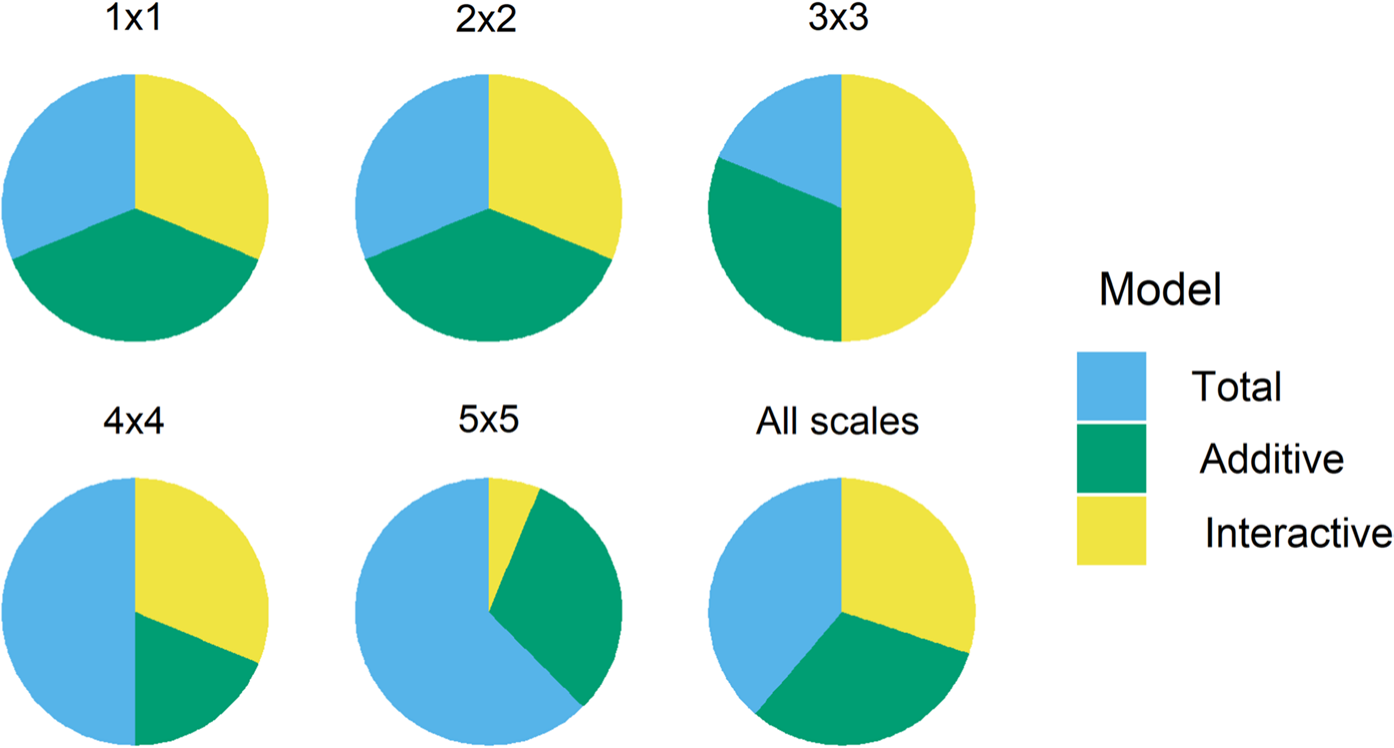
Proportion of species for which each model type received the highest model weight among twelve bird species at five spatial scales. Models estimated the cumulative effect of anthropogenic disturbance (oil and gas footprint) on occupancy rate. *Total* all footprint types combined, *Additive* independent effects of footprint types, *Interactive* all pairwise interactions of footprint types. Footprint types were: seismic lines; wide linear disturbances (e.g. roads, powerlines); well pads; and industrial sites. Model weights were calculated as pseudo Bayesian Model Averaging weights

Model comparison across species and scales showed even representation among model types at small scales (1 × 1 and 2 × 2), while total footprint models become dominant at the largest scales (4 × 4 and 5 × 5) (Fig. 6). There was a strong negative relationship between spatial scale and model selection uncertainty, with best models for 12 of 16 species having < 50% of model weight at the 1 × 1 scale, and 15 of 16 having > 90% of model weight at the 5 × 5 scale (Table 2). Mean weight of the top model increased steadily with scale, being 0.46, 0.66, 0.89, 0.92, and 0.95 for the 1 × 1, 2 × 2, 3 × 3, 4 × 4, and 5 × 5 scales, respectively. We found few strong relationships between footprint variables and occupancy rates at any scale. The most consistent variable was total footprint at the two largest scales. We found one case where the effect of a variable changed between scales (Swainson’s Thrush, Table 2). Ten species showed moderate or strong positive relationships between footprint variables and occupancy rates at the 4 × 4 and 5 × 5 scales (Table 2). Of these, seven were associated with open habitats (Lincoln’s Sparrow, Alder Flycatcher, Clay-colored Sparrow), or recently disturbed areas (Tennessee Warbler, Mourning Warbler, Magnolia warbler, Chipping Sparrow) in Alberta.

We found evidence for domains of scale in our visual inspections of plots and non-parametric tests of parameter estimates among scales. Visual inspection showed clear, consistent patterns among plots in distributions of parameter estimates among species and across scales. Specifically, we saw the strongest relationships at the 4 × 4 and 5 × 5 scales, marginally strong relationships at the 1 × 1 scale, and all relationships near zero at the 2 × 2 and 3 × 3 scales, across all model types (Fig. 5, A–C). Our non-parametric tests found evidence for statistically significant differences among scales in parameter estimates (Kruskal-Wallace: ${\chi }^{2}=22.2,df=4,p<0.001$). The Wilcoxon test showed mean parameter estimates were not different among scales 1 × 1–3 × 3, and among scales 4 × 4–5 × 5 but that these two groups were significantly different from each other ($p<0.05$). Pairing these two sets of results suggests domains of scale at 1 × 1 and 4 × 4–5 × 5, with 2 × 2–3 × 3 acting as a transition zone.

## Discussion

Our analysis showed that, while occupancy probability of some bird species is affected by oil and gas footprint in the oil sands region of Alberta, these effects are not constant across spatial scales. The overall pattern across species and scales provides very strong evidence for domains of scale in the response of territorial passerines to energy sector footprint. Our study is one of the first to examine scale continua for establishing domains of scale in the relationship between species distributions and environmental conditions (but see Wheatley & Larsen 2018). The novel sampling design and modeling framework employed here represent a major advance in incorporating scale dependencies in modelling species responses to disturbance. Our results provide a rigorous quantitative evaluation of the complex interactions among habitat selection, landscape disturbance, and spatial scale of ecological processes in determining effects of multiple stressors on boreal birds. Given the dominant role of observational scale in results of ecological investigations, our work provides an important new framework for linking local and landscape scale processes to cumulative effects of anthropogenic disturbance on wildlife populations.

Although the fundamental role of scale in landscape ecology is well established (McGarigal et al. 2016), empirical studies that incorporate scale directly into the study design are almost non-existent (Wheatley & Larsen 2018). Our sampling design is a variation of the scale continuum approach applied by Wheatley & Larsen (2018), but incorporates multiple hierarchical levels into the same model. In our case, while the point-level habitat selection scale remained constant, the block-level scale followed a continuum where both the response (block-level occupancy) and predictor (footprint) variables scaled accordingly. This continuum approach is what allowed us to directly compare model coefficients across multiple scales, thus providing the ability to identify scale domains in species’ responses to environmental predictors.

Our modeling framework is unique relative to previously published hierarchical occupancy models (Hines et al. 2010; Mordecai et al. 2011; Crosby & Porter 2018; e.g. Nichols et al. 2008). In contrast to previous models, where sub-sample occupancy is conditional on sample unit occupancy probability, we condition point-level occupancy on the probability that a given point within the block is occupied. The effect of this difference is that the model is estimating footprint effects on within-unit occupancy rate (as opposed to unit-level occupancy probability), thereby making it independent of scale, and so allowing for direct comparisons across scales. In contrast, estimating occupancy probability at the sample unit level means probability automatically increases with sample unit extent, so that estimates are not comparable across scales. This framework also allowed us to make inferences about cumulative effects of footprint independent of local habitat effects. Doing so creates a more realistic estimate of how local vegetation affects subsample occupancy probability, thereby explicitly separating it from effects of variables of interest measured at larger spatial scales.

Our study is the first to find evidence for multiple scale domains representing individual- and population-level processes, as well as a potential transition zone between the domains (Fig. 5). The existence of such domains in the process of habitat selection and species distributions, and potential reasons for the pattern, have been hypothesized for quite some time (Wiens 1989; Holling 1992; Bestelmeyer et al. 2003), but seen little investigation. As a rare example, Fisher et al. (2011) found evidence for allometric scaling in the spatial scales that best describe habitat selection in mammals, concluding that these optimal scales represented species-specific scale domains for the process of habitat selection. In a similar example, Wheatley & Larsen (2018) found evidence for potential scale domains in habitat selection in northern flying squirrels (*Glaucomys sabrinus*). The evidence for multiple scale domains revealed by our analysis are congruent with the links to processes proposed by Bestelmeyer et al. (2003). More specifically, apparent scale domains at the 1 × 1 and 4 × 4–5 × 5 scales appear to correspond to the habitat selection and landscape distribution domains, respectively (sensu Bestelmeyer et al. 2003). The ability to investigate scale domains representing local- and landscape-scale processes simultaneously was a function of our unique sampling design and modelling framework, and represents an advancement in our ability to explicitly incorporate scale into ecological research.

The sampling design and modelling framework developed in this research addresses one aspect of what is known in geography as the modifiable areal unit problem (MAUP, Manley 2021). The MAUP occurs when results of analyses can vary depending on the zonation (shape and orientation) and spatial scale of data aggregation (Jelinski & Wu 1996). The MAUP has been considered primarily in physical geography and the social sciences, typically in the arbitrary aggregation of data into census tracts or political boundaries, but has seen little treatment in landscape ecology (Jelinski & Wu 1996; Boyce et al. 2017). Our research directly addresses the scale aspect of the MAUP by allowing us to compare results across scales. Although the MAUP is generally considered to be a statistical issue related to sampling design, rather than an ecological issue, our research shows that in ecology the two cannot necessarily be separated.

Other studies in this region have reported large effects of footprint on breeding bird densities (Bayne et al. 2016; Mahon et al. 2019) and relative abundance of mammals (Fisher & Burton 2018; Toews et al. 2018). These studies provide strong evidence for local effects of footprint in the immediate area of disturbance. Of these, Mahon et al. (2019) was the first to examine interacting effects of multiple stressors at larger spatial scales, and was most comparable to our 3 × 3 scale of analysis. We found more limited evidence for footprint effects on birds than either Mahon et al. (2019) or Bayne et al. (2016), and the differences can largely be explained by study design. One important difference between these analyses is that Mahon et al. (2019) did not separate local habitat effects from landscape-scale footprint, but rather averaged both over the entire sampling unit. A potential effect of this design may have been to dilute the effect of point-scale local habitat and so attribute a greater portion of variability to footprint. Our analysis, by contrast, estimated point-level habitat effects identical to Sólymos et al. (2020), assuming habitat was the primary driver of point-level occupancy, and could be modified by oil and gas footprint at the landscape scale. Another major difference is that Mahon et al. (2019) included forest harvest units as part of the footprint because both energy sector and forestry sector disturbances occurred in a non-uniform distribution created by different temporal and spatial patterns (e.g. the timing, type, intensity, and location) of development within a large, multi-sector landscape. This likely increased the potential to find both individual effects and interactions. Our analysis, on the other hand, only examined energy sector footprint and assumed forestry and natural disturbance had the same effect on occupancy through forest age. While we acknowledge this assumption is not necessarily true in many cases, the fact that we were not concerned with forestry effects makes it consistent with our objectives, and we do not feel it affected our conclusions about energy sector effects on birds.

Bayne et al (2016) used disturbance type as a categorical variable at spatial scales smaller than, or roughly equivalent to, our point-level habitat variables (0–50 m, 0–100 m, 0–unlimited). Although we did not explicitly include footprint at the point level, the down-weighting of dominant habitat for total area covered likely accounted somewhat for potential local footprint effects on occupancy. Like our point-level data, in the analyses of Mahon et al. (2019), the fact that habitat and footprint were both measured as proportion of land cover throughout the sample unit meant that as footprint increased, proportion of area measured as any habitat type decreased within the sample unit, automatically causing it to weigh more heavily relative to habitat in terms of influence on sample unit bird density. Because we used dominant habitat weighted for proportion of area covered as our point-level occupancy predictor, the habitat effect in our models was not conditional on footprint, except insofar as footprint within 150 m would reduce the weight of habitat at that point.

Most of the strong bird-footprint relationships we observed were positive, and reflect local colonization by generalist or non-forest species, consistent with Bayne et al. (2016). These species are not strongly associated with natural disturbance processes like fire, consistent with studies showing energy sector development is driving recent shifts in anthropogenic disturbance patterns away from patterns produced by natural disturbance (Pickell et al. 2015). It is notable that our analysis did not find many negative relationships between footprint and occupancy rates, particularly for species known to be sensitive to disturbance. A potential explanation for this is non-linearity in the occupancy-abundance relationship (Freckleton et al. 2005). There is generally a reliably positive relationship between occupancy and abundance (Zuckerberg et al. 2009). However, this relationship may become unstable due to Allee effects, dispersal probabilities, or spatio-temporal sampling scale (Freckleton et al. 2005; Steenweg et al. 2018). Birds are highly mobile and tend to colonize new areas easily, and species with larger populations tend to be buffered against local extinctions (Freckleton et al. 2005). The fact that we limited our analysis to species detected at $\ge$ 10% of point count stations (except Blue-headed Vireo, detected at 9.2% of stations) could have made finding negative effects at larger scales less likely. It is possible that including more uncommon species would have shown more negative responses to footprint at larger scales. Future analyses that include a wider range of species could help shed more light on this issue.

It is notable that, as scale increased, total area of energy sector footprint became the dominant predictor among species. At the 5 × 5 scale, total footprint area was the top model for ten of sixteen species, and among these, the average pBMA model weight was 0.999. It could be argued that, as scale increased, the likelihood of seeing additive and interactive effects should increase as well because the probability of having multiple footprint types would be higher, however this was not the case. This strong and consistent pattern, combined with the positive relationship with open habitat and disturbance-dependent species, suggests a link between total footprint and the potential for biotic homogenization at these larger scales. Future research should investigate this important implication, as the mechanisms that maintain or alter regional biodiversity are scale-dependent, and understanding these mechanisms is important for making landscape-specific conservation decisions (Socolar et al. 2016).

A critical point about the sampling and analysis framework presented here is that it illustrates scale-dependence in ecological interpretations. For example, an analysis conducted only at the largest scales would have missed the highly variable effects of human footprint on individual habitat selection. Conversely, attempting to scale up results from the 1 × 1 scale would have resulted in very different patterns than what we found at the 4 × 4 and 5 × 5 scales. This inability to extrapolate results across scales is one of the most long standing problems in landscape ecology (Turner et al. 1989; Wiens 1989). One implication of this is that models developed at different spatial scales will not necessarily scale up the same way when being projected to regional extents, creating large uncertainties in population estimates and distributional patterns. Understanding why patterns change at different scales is the key to linking predictions across scale domains (Wheatley & Larsen 2018), and so should be a major focus of future research in cumulative effects assessment.

## Conclusions

Our evidence for scale domains clearly demonstrates the risk in assuming that patterns observed at one scale will be predictive of either the smaller scale processes driving those patterns, or of patterns that will emerge at larger scales. We found evidence that effects of anthropogenic disturbance on bird populations are manifested primarily at the point scale as a result of the habitat selection process, but that landscape scale distributional effects become dominant as sampling unit extent increases. Our results strongly suggest that when developing models of cumulative effects, it is necessary to view the landscape at multiple spatial scales in order to incorporate different processes operating to determine species’ distributions and abundances. For management purposes, planning and regulation should be done over a range of organizational levels, representing hierarchically nested spatial scales, to reflect the scale domains of different ecological processes appropriate to the species and management questions of interest. Such hierarchically structured planning is necessary if biodiversity is to be maintained on landscapes that are heavily altered by industrial development.

### Supplementary Information

Below is the link to the electronic supplementary material.

Supplementary file1 (DOCX 21 KB)

Supplementary file2 (DOCX 22 KB)

## Data Availability

Raw avian data is publicly available on the WildTrax website: https://www.wildtrax.ca/home/. Raw habitat and human footprint data is proprietary data owned by Alberta Biodiversity Monitoring Institute: https://abmi.ca/home.html. Post-processed data used to fit the models is archived at https://github.com/crosbya1/multi-level-occupancy/blob/main/docs/big-grid-data.Rdata.

## References

[CR1] Alberta Biodiversity Monitoring Institute. (2019). Wall to wall human footprint inventory. Alberta Biodiversity Monitorint Institute, Geospatial Centre. https://ftp-public.abmi.ca/GISData/HumanFootprint/2017/HFI2017v22_Metadata.pdf.

[CR2] Bayne EM, Leston L, Mahon CL, Sólymos P, Machtans C, Lankau H, Ball JR, Van Wilgenburg SL, Cumming SG, Fontaine T, Schmiegelow FKA, Song SJ (2016). Boreal bird abundance estimates within different energy sector disturbances vary with point count radius. The Condor.

[CR3] Bestelmeyer BT, Miller JR, Wiens JA (2003). Applying species diversity theory to land management. Ecol Appl.

[CR4] Blancher, P., & Wells, J. (2005). The boreal forest region: North America’s bird nursery. Boreal Songbird Initiative. https://www.borealbirds.org/sites/default/files/publications/report-bsi-birdnursery.pdf.

[CR5] Boyce MS, Mallory CD, Morehouse AT, Prokopenko CM, Scrafford MA, Warbington CH (2017). Defining landscapes and scales to model landscape-organism interactions. Curr Landscape Ecol Rep.

[CR6] Charchuk C, Bayne EM (2018). Avian community response to understory protection harvesting in the boreal forest of Alberta, Canada. For Ecol Manage.

[CR7] Crosby AD, Porter WF (2018). A spatially explicit, multi-scale occupancy model for large-scale population monitoring. J Wildl Manag.

[CR8] Ecological Stratification Working Group. (1995). A national ecological framework for Canada. Agriculture and Agri‐Food Canada, Research Branch, Centre for Land and Biological Resources Research and Environment Canada, State of the Environment Directorate. Cat. No. A42–65/1996E; ISBN 0–662–24107-X

[CR9] Fisher JT, Burton AC (2018). Wildlife winners and losers in an oil sands landscape. Front Ecol Environ.

[CR10] Fisher JT, Anholt B, Volpe JP (2011). Body mass explains characteristic scales of habitat selection in terrestrial mammals. Ecol Evol.

[CR11] Freckleton RP, Gill JA, Noble D, Watkinson AR (2005). Large-scale population dynamics, abundance-occupancy relationships and the scaling from local to regional population size. J Anim Ecol.

[CR12] Gerber BD, Kendall WL, Hooten MB, Dubovsky JA, Drewien RC (2015). Optimal population prediction of sandhill crane recruitment based on climate-mediated habitat limitations. J Anim Ecol.

[CR13] Hines JE, Nichols JD, Royle JA, MacKenzie DI, Gopalaswamy AM, Kumar NS, Karanth KU (2010). Tigers on trails: occupancy modeling for cluster sampling. Ecol Appl.

[CR14] Höge M, Guthke A, Nowak W (2020). Bayesian model weighting: The many faces of model averaging. Water (switzerland).

[CR15] Holling CS (1992). Cross-scale morphology, geometry, and dynamics of ecosystems. Ecol Monogr.

[CR16] Hooten MB, Hobbs NT (2015). A guide to Bayesian model selection for ecologists. Ecol Monogr.

[CR17] Alberta Biodiversity Monitoring Institute. (2017). Alberta wall-to-wall vegetation layer including “backfilled” vegetation in human footprints (Version 6). Alberta Biodiversity Monitoring Institute. https://www.abmi.ca/home/data-analytics/da-top/da-product-overview/Data-Archive/Detailed-Vegetation-Maps.html.

[CR18] Jelinski DE, Wu J (1996). The modifiable areal unit problem and implications for landscape ecology. Landscape Ecol.

[CR19] Kellner, K. F. (2015). jagsUI: a wrapper around rjags to streamline JAGS analyses. https://cran.r-project.org/web/packages/jagsUI/index.html.

[CR20] Leston L, Bayne E, Toms JD, Mahon CL, Crosby A, Sólymos P, Ball J, Song SJ, Schmiegelow FKA, Stralberg D, Docherty TDS (2023). Comparing alternative methods of modelling cumulative effects of oil and gas footprint on boreal bird abundance. Landscape Ecol.

[CR21] Mahon CL, Pelech S (2021). Guidance for analytical methods to cumulative effects assessment for terrestrial species. Environ Rev.

[CR22] Mahon CL, Holloway G, Sólymos P, Cumming SG, Bayne EM, Schmiegelow FKA, Song SJ (2016). Community structure and niche characteristics of upland and lowland western boreal birds at multiple spatial scales. For Ecol Manage.

[CR23] Mahon CL, Holloway GL, Bayne EM, Toms JD (2019). Additive and interactive cumulative effects on boreal landbirds: winners and losers in a multi-stressor landscape. Ecol Appl.

[CR24] Manley D, Fischer MM, Nijkamp P (2021). Scale, Aggregation, and the Modifiable Areal Unit Problem. Handbook of Regional Science.

[CR25] McGarigal K, Wan HY, Zeller KA, Timm BC, Cushman SA (2016). Multi-scale habitat selection modeling: a review and outlook. Landscape Ecol.

[CR26] Mordecai RS, Mattsson BJ, Tzilkowski CJ, Cooper RJ (2011). Addressing challenges when studying mobile or episodic species: hierarchical Bayes estimation of occupancy and use. J Appl Ecol.

[CR27] Nichols JD, Bailey LL, A. F. O., Talancy, N. W., Grant, E. H. C., Gilbert, A. T., Annand, E. M., Husband, T. P., & Hines, J. E. (2008). Multi-scale occupancy estimation and modelling using multiple detection methods. J Appl Ecol.

[CR28] Olden JD (2006). Biotic homogenization: a new research agenda for conservation biogeography. J Biogeogr.

[CR29] Pickell PD, Andison DW, Coops NC, Gergel SE, Marshall PL (2015). The spatial patterns of anthropogenic disturbance in the western Canadian boreal forest following oil and gas development. Can J for Res.

[CR30] Plummer, M. (2003). JAGS: A program for analysis of Bayesian graphical models using Gibbs sampling (K. Hornik, F. Leisch, & A. Zeileis, Eds.). DSC 2003. http://www.ci.tuwien.ac.at/Conferences/DSC-2003/.

[CR31] R Core Development Team. (2017). R: A language and environment for statistical computing. R Foundation for Statistical Computing. https://www.r-project.org/.

[CR32] Socolar JB, Gilroy JJ, Kunin WE, Edwards DP (2016). How should beta-diversity inform biodiversity conservation?. Trends Ecol Evol.

[CR33] Sólymos P, Toms JD, Matsuoka SM, Cumming SG, Barker NKS, Thogmartin WE, Stralberg D, Crosby AD, Dénes FV, Haché S, Mahon CL, Schmiegelow FKA, Bayne EM (2020). Lessons learned from comparing spatially explicit models and the Partners in Flight approach to estimate population sizes of boreal birds in Alberta, Canada. The Condor.

[CR34] Steenweg R, Hebblewhite M, Whittington J, Lukacs P, McKelvey K (2018). Sampling scales define occupancy and underlying occupancy-abundance relationships in animals. Ecology.

[CR35] Stevens BS, Conway CJ (2019). Predicting species distributions: unifying model selection and scale optimization for multi-scale occupancy models. Ecosphere.

[CR36] Toews M, Juanes F, Burton AC (2018). Mammal responses to the human footprint vary across species and stressors. J Environ Manage.

[CR37] Turner MG, Dale VH, Gardner RH (1989). Predicting across scales: Theory development and testing. Landscape Ecol.

[CR38] Vehtari, A., Gabry, J., Magnusson, N., Yao, Y., Bürkner, P., Paananen, T., & Gelman, A. (2020). loo: Efficient leave-one-out cross-validation and WAIC for Bayesian models. https://mc-stan.org/loo/.

[CR39] Venier LA, Thompson ID, Fleming R, Malcolm J, Aubin I, Trofymow JA, Langor D, Sturrock R, Patry C, Outerbridge RO, Holmes SB, Haeussler S, Grandpré LD, Chen HYH, Bayne E, Arsenault A, Brandt JP (2014). Effects of natural resource development on the terrestrial biodiversity of Canadian boreal forests. Environ Rev.

[CR40] Venier LA, Walton R, Brandt JP (2021). Scientific considerations and challenges for addressing cumulative effects in forest landscapes in Canada. Environ Rev.

[CR41] Watanabe, S. (2010). Asymptotic Equivalence of Bayes Cross Validation and Widely Applicable Information Criterion in Singular Learning Theory. https://www.jmlr.org/papers/volume11/watanabe10a/watanabe10a.pdf.

[CR42] Watson JEM, Evans T, Venter O, Williams B, Tulloch A, Stewart C, Thompson I, Ray JC, Murray K, Salazar A, McAlpine C, Potapov P, Walston J, Robinson JG, Painter M, Wilkie D, Filardi C, Laurance WF, Houghton RA, Lindenmayer D (2018). The exceptional value of intact forest ecosystems. Nat Ecol Evol.

[CR43] Wheatley M (2010). Domains of scale in forest-landscape metrics: Implications for species-habitat modeling. Acta Oecologica.

[CR44] Wheatley M, Larsen K (2018). Scale relativity of species-habitat models. Ecol Complex.

[CR45] Wiens JA (1989). Spatial scaling in ecology. Funct Ecol.

[CR46] Zuckerberg B, Porter WF, Corwin K (2009). The consistency and stability of abundance-occupancy relationships in large-scale population dynamics. J Anim Ecol.

