# Supplementary material for: Domains of scale in cumulative effects of energy sector development on boreal birds
Source: Landsc Ecol. 2023 Oct 25;38(12):3173–88. doi: 10.1007/s10980-023-01779-8 (PMC10754738; doi:10.1007/s10980-023-01779-8)
Supplement: Supplementary file 1 — Supplementary file1 (DOCX 21 KB) [file 10980_2023_1779_MOESM1_ESM.docx]

# **Appendix A: Vegetation and human footprint categories**

**Table 1**: Vegetation types from the Alberta Biodiversity Monitoriing Institute (ABMI) wall-to-wall vegetation database used for modeling breeding bird occupancy probability in northern Alberta, Canada.

| Vegetation type | Habitat type | Area (ha) | Percentage  of area |
| --- | --- | --- | --- |
| Deciduous forest | Deciduous | 14,949 | 27.7 |
| Mixedwood forest | Mixed | 3,108 | 5.8 |
| Conifer forest | Pine | 5,786 | 10.7 |
| Upland spruce forest | Spruce | 3,251 | 6.0 |
| Treed bog | Black spruce | 8,525 | 15.8 |
| Treed fen | Larch | 6,671 | 12.4 |
| Treed swamp | Larch | 555 | 1.0 |
| Graminoid fen | Lowland | 844 | 1.6 |
| Marsh | Lowland | 293 | 0.5 |
| Shrubby bog | Lowland | 173 | 0.3 |
| Shrubby fen | Lowland | 644 | 1.2 |
| Shrubby swamp | Lowland | 1,010 | 1.9 |
| Open water | Lowland | 1,383 | 2.6 |
| Upland grass/herb | Open | 115 | 0.2 |
| Upland shrub | Open | 540 | 1.0 |
| Bare ground | Open | 0 | 0.0 |
| Snow/ice |  | 0 | 0.0 |

**Table 2**: Anthropogenic disturbance types from the Alberta Biodiversity Monitoriing Institute (ABMI) wall-to-wall human footprint database used for modeling breeding bird occupancy probability in northern Alberta, Canada.

| Footprint type | Footprint class | Area (ha) | Percentage  of area |
| --- | --- | --- | --- |
| Seismic line wide | Seismic | 403 | 0.7 |
| Seismic line narrow | Seismic | 595 | 1.1 |
| Pipeline | Wide linear | 854 | 1.6 |
| Transmission line | Wide linear | 134 | 0.2 |
| Road: vegetated | Wide linear | 194 | 0.4 |
| Road: vegetated verge | Wide linear | 549 | 1.0 |
| Railroad verge | Wide linear | 25 | 0.0 |
| Road: hard surface | Wide linear | 240 | 0.4 |
| Rail: hard surface | Wide linear | 8 | 0.0 |
| Well site | Well | 1,373 | 2.5 |
| Industrial site rural | Industry | 1,497 | 2.8 |
| Mine site | Industry | 52 | 0.1 |
| Man-made water body | Industry | 138 | 0.3 |
| Other | Industry | 14 | 0.0 |
