# Supplementary material for: Domains of scale in cumulative effects of energy sector development on boreal birds
Source: Landsc Ecol. 2023 Oct 25;38(12):3173–88. doi: 10.1007/s10980-023-01779-8 (PMC10754738; doi:10.1007/s10980-023-01779-8)
Supplement: Supplementary file 2 — Supplementary file2 (DOCX 22 KB) [file 10980_2023_1779_MOESM2_ESM.docx]

#### Appendix B: Running a multi-level occupancy model with Bayesian lasso for variable selection

# Overview

This is Appendix B from the manuscript “Domains of scale in cumulative effects of energy sector development on boreal birds” by Crosby et al. (n.d.). submitted to the journal *Landscape Ecology*. The objective of this document is to describe, and demonstrate how to run, the scale integrated occupancy model using Bayesian lasso for variable selection.

This file is avaialable as a README file in the Github repository: <https://github.com/crosbya1/multi-level-occupancy/tree/main>

## **Software requirements:**

- R for downloading and processing data, and running the models (R Core Development Team, 2017), downloadable from <https://www.r-project.org/>
- JAGS for estimating model parameters using Markov chain Monte Carlo (MCMC) through the use of a Gibbs sampler (Plummer, 2003), downloadable from <https://mcmc-jags.sourceforge.io/>
- R extension packages: **tidyverse**, **jagsUI**, and **loo**. These can be installed from R using the “install.packages” function

## **Files:**

- **big-grid-data.Rdata**: Post-processed data used to fit the models in this analysis
  - These data can be downloaded from the Github repository at: [big-grid-data.Rdata](https://github.com/crosbya1/multi-level-occupancy/blob/main/docs/big-grid-data.Rdata)

To be consistent with the [GitHub repository](https://github.com/crosbya1/multi-level-occupancy), when following this tutorial in R, create a directory named “docs” within your R working directory to store the data in.

# Importing and processing the Big Grid data

We will run this example on a small amount of data, using Ovenbird (*Seiurus aurocapilla*), using 20 blocks at the 2x2 scale.

library(tidyverse)

# Load the data
load("docs/big-grid-data.Rdata")

# Select the species
spp_code <- "OVEN"

# specify the number of blocks
nblock <- 20

# Specify the 2x2 scale.
scale <- 2

# Get the species detection data
spp_point <- bg_pa[, c(1:2, which(colnames(bg_pa) == spp_code))]

# Reformat the data to 'wide form'
spp_pt <- spp_point %>% spread(survey, spp_code)
spp_pt <- spp_pt[match(bg_nsurv_veg$SS, spp_pt$SS), 1:5]

# Create the test dataset
set.seed(1234)
test <- sort(sample(1:nrow(blocks_veghf_2[[scale]]), nblock, replace = FALSE))
block.test <- blocks_veghf_2[[scale]][test, ]

pt.test <- point.block[[scale]][which(point.block[[scale]]$block %in% block.test$blockID), ]

# Get the dominant vegetation and weighted mean forest age for each point
pt_cov.test <- do.call(rbind, lapply(1:nlevels(as.factor(pt.test$block)), function(x){
 d <- pt.test[which(pt.test$block == levels(as.factor(pt.test$block))[x]), ]
 d1 <- data.frame(d, veghf_dom[match(d$ss, rownames(veghf_dom)), ])
 return(d1)
}))

# Get the grid, point, and block information for each point
pt_block.test <- pt_cov.test[, c("ss", "grid", "point", "block")]

# Get the detection covariates
det_cov.test <- array(NA, dim = c(nrow(pt_block.test), dim(det.var_veg)[2:3]))
dimnames(det_cov.test) <- list(pt_block.test$ss, dimnames(det.var_veg)[[2]], dimnames(det.var_veg)[[3]])
for(i in 1:nrow(pt_cov.test)){
 d <- which(rownames(det.var_veg) == rownames(det_cov.test)[i])
 det_cov.test[i, , ] <- det.var_veg[d, , ]
}

# Get the detection data and number of surveys for the points included in the analysis
spp.test <- do.call(rbind, lapply(1:nrow(pt_cov.test), function(x) spp_pt[which(spp_pt$SS == pt_cov.test$ss[x]), ]))

nsurv.test <- do.call(rbind, lapply(1:nrow(pt_cov.test), function(x) bg_nsurv_veg[which(bg_nsurv_veg$SS == pt_cov.test$ss[x]), ]))
all.equal(nsurv.test$SS, pt_block.test$ss) # Make sure the data mathces up


# Get the human footprint data for the blocks included in the analysis
block_cov.test <- as.matrix(block.test[, grep("seismic|widelin|wells|industry", colnames(block.test))])


save(block.test, block_cov.test, pt_cov.test, spp_pt, spp.test, det.var_veg, det_cov.test, nsurv.test, file = "docs/lasso_data.Rdata")

# Description of model code

## **Hierarchical model code**

# This model estimates point-level occupancy probability as a joint
# probability of block-level human footprint and point-level habitat,
# where blocks contain >= 1 point.

sink("block_occupancy_lasso.txt")
cat("
 model{

 #Priors on block-level coefficients (for Bayesian Lasso, uses double exponential distribution)
 beta.block[1] ~ dnorm(0, 1)
 for(i in 2:n.beta.block){
 beta.block[i] ~ ddexp(0, lambda) # where lamdba is the double exponential variance parameter specified for each Bayesian Lasso iteration
 }

 #Priors on point-level coefficients
 beta.point[1] ~ dnorm(0, 1)
 for(i in 2:n.beta.point){
 beta.point[i] ~ dnorm(0, 0.1)
 }

 # Priors on detection coefficients
 for(i in 1:n.beta.p){
 beta.p[i] ~ dnorm(0, 0.1)
 }


 # Likelihood on mean within-block occupancy probability
 for(i in 1:n.block){
 logit(psi[i]) <- inprod(beta.block, cov.block[i, ]) # block-level occupancy probability
 delta[i] <- 1 - pow((1 - psi[i]), (1/n.scale)) # the proportion of area occupied as a function of human footprint / constant point-level occupancy probability
 N[i] ~ dbinom(delta[i], n.scale) # Number of occupied points as a function of block-level human footprint
 }

 # Likelihood point-level occupancy as a function of habitat, conditional on block-level occupancy rate
 for(i in 1:n.site){
 zb[i] ~ dbern(delta[point.block[i]]) # Point-level occupancy as a function of block-level parameters
 logit(theta[i]) <- inprod(beta.point, cov.point[i, ])
 zp[i] ~ dbern(zb[i]*theta[i])
 # Likelihood on detecting the species conditional on occupancy
 for(j in 1:n.surv[i]){
 logit(p[i, j]) <- inprod(beta.p, cov.p[i, j, ])
 y[i, j] ~ dbern(zp[i]*p[i, j])

 prob1[i, j] <- pow(p[i, j], y[i, j])*pow((1-p[i, j]), (1 - y[i, j]))
 prob2[i, j] <- 1 - p[i, j]
 }
 # Model likelihood calculation
 term1[i] <- indicator[i]*(psi[point.block[i]]*theta[i])*prod(prob1[i, 1:n.surv[i]])
 term2[i] <- (1 - indicator[i])*((1 - (psi[point.block[i]]*theta[i])) + (psi[point.block[i]]*theta[i])*prod(prob2[i, 1:n.surv[i]]))
 prob.y[i] <- term1[i] + term2[i]
 lprob.y[i] <- log(prob.y[i])

 # Calculate the probability of detecting it at least once,
 prob.i[i] <- (zp[i]*theta[i])*(1 - prod(1 - p[i, 1:n.surv[i]])) # Probability of occupancy * probability of detecting at least once
 zc[i] ~ dbern(prob.i[i])
 }

 #Derived quantities
 l.score <- -2*sum(lprob.y[]) # Model likelihood estimate
 e.count.total <- sum(zc[]) # Total number of occupied points
}
", fill = TRUE)
sink()

For implementation in JAGS, we specified the double exponential distribution as the prior distribution for all block-level variables as:

‘beta.block[i] ~ ddexp(0, lambda)’ (see model code above), where lambda was the prior variance value for each realization of the model.

# Bayesian Lasso implementation

## **Details on Bayesian Lasso implementation**

We implemented the Bayesian Lasso procedure for coefficients on the block-level portion of the model, with specific variables depending on which model type was being estimated. Similar to Gerber et al. (2015) and Stevens and Conway (2019), we searched over 50 potential values of prior precision for the Laplace distribution ranging from 0.1–5 on the log scale, which translated to ~1.1–148 when transformed. We selected the optimal prior for each model type at each scale by comparing models using Watanabe-Akaike Information Criterion (WAIC, Watanabe, 2010).

For this demonstration, we will limit the lasso implementation to 10 values of the prior variance, as the models are run separately for each value of the variance. For simplicity, we will use total human footprint as the only block-level covariate. In the full analysis, these models were run in parallel on a high performance computing system (Digital Research Alliance of Canada, formerly Compute Canada).

# Load the libraries
library(jagsUI)
library(loo)

# Load the data
load("docs/lasso_data.Rdata")

block.cov <- data.frame(total.hf = rowSums(block_cov.test))

# Create the block-level model matrix
cov.block <- model.matrix(~ ., data = data.frame(block.cov))

# Create the point-level model matrix, with forest age scaled
pt.cov <- data.frame(age = scale(pt_cov.test$age), pt_cov.test[, grep("dom", colnames(pt_cov.test))])
cov.point <- model.matrix(~ as.matrix(pt.cov))

# Create the array of detection covariates
cov.p <- det_cov.test[, , 1:3]

# Set the lambda values for lasso testing
nlam <- 10
log.lambda=seq(0.1, 5, length = nlam)
lambda <- exp(log.lambda)

First, run a single model with the lowest lambda value

scale <- 2
ni <- 1000
nb <- 500
nt <- 1
nc <- 3


mod.lam <- lambda[1] # The lambda value to use


params_1 <- c("beta.block", "beta.point", "beta.p", "l.score", "lprob.y")
zst <- apply(spp.test[, -1], 1, function(x) max(x, na.rm = T))
zbst <- rep(1, nrow(cov.point))

inits <- function() {list(zp = zst, zb = zbst, beta.block = rnorm(ncol(cov.block)), beta.point = rnorm(ncol(cov.point)), beta.p = rnorm(dim(cov.p)[3]))}

data <- list(y = spp.test[, -1], cov.p = cov.p, cov.point = cov.point, cov.block = cov.block, n.surv = nsurv.test$nsurv, n.site = nrow(spp.test), n.block = nrow(cov.block), n.beta.block = ncol(cov.block), n.beta.point = ncol(cov.point), n.beta.p = dim(cov.p)[3], n.scale = scale^2, block = as.numeric(as.factor(block.test$blockID)), point.block = as.numeric(as.factor(pt_cov.test$block)), indicator = zst, lambda = mod.lam)

system.time({
 out <- jags(data = data, inits = inits, parameters.to.save = params_1,
 model.file = "block_occupancy_lasso.txt", n.chains = nc, n.thin = nt,
 n.iter = ni, n.burnin = nb, parallel = TRUE)
})

Next, put it into a loop and save the log scores to find the lambda values that best fit the data

lasso_scores <- lapply(1:length(lambda), function(i){
 mod.lam <- lambda[i] # The lambda value to use


 params_1 <- c("beta.block", "beta.point", "beta.p", "l.score", "lprob.y")
 zst <- apply(spp.test[, -1], 1, function(x) max(x, na.rm = T))
 zbst <- rep(1, nrow(cov.point))

 inits <- function() {list(zp = zst, zb = zbst, beta.block = rnorm(ncol(cov.block)), beta.point = rnorm(ncol(cov.point)), beta.p = rnorm(dim(cov.p)[3]))}

 data <- list(y = spp.test[, -1], cov.p = cov.p, cov.point = cov.point, cov.block = cov.block, n.surv = nsurv.test$nsurv, n.site = nrow(spp.test), n.block = nrow(cov.block), n.beta.block = ncol(cov.block), n.beta.point = ncol(cov.point), n.beta.p = dim(cov.p)[3], n.scale = scale^2, block = as.numeric(as.factor(block.test$blockID)), point.block = as.numeric(as.factor(pt_cov.test$block)), indicator = zst, lambda = mod.lam)

 system.time({
 out <- jags(data = data, inits = inits, parameters.to.save = params_1,
 model.file = "block_occupancy_lasso.txt", n.chains = nc, n.thin = nt,
 n.iter = ni, n.burnin = nb, parallel = TRUE)
 })

 Rhats <- out$Rhat$beta.block
 log.score <- out$mean$l.score
 waic <- waic(out$sims.list$lprob.y)

 return(list(waic = waic$estimates[3, 1], log.score = log.score, Rhats = Rhats))

})

Crosby, A. D., Leston, L., Bayne, E. M., Sólymos, P., Mahon, C. L., Toms, J. D., Docherty, T. D. S., & Song, S. J. (n.d.). *Domains of scale in cumulative effects of energy sector development on boreal birds*. https://doi.org/<https://doi.org/10.21203/rs.3.rs-2191857/v1>

Gerber, B. D., Kendall, W. L., Hooten, M. B., Dubovsky, J. A., & Drewien, R. C. (2015). Optimal population prediction of sandhill crane recruitment based on climate-mediated habitat limitations. *Journal of Animal Ecology*, *84*(5), 1299–1310. <https://doi.org/10.1111/1365-2656.12370>

Plummer, M. (2003). *JAGS: Just another gibbs sampler*. <http://mcmc-jags.sourceforge.net/>

R Core Development Team. (2017). *R: A language and environment for statistical computing*. R Foundation for Statistical Computing. <https://www.r-project.org/>

Stevens, B. S., & Conway, C. J. (2019). Predicting species distributions: unifying model selection and scale optimization for multi‐scale occupancy models. *Ecosphere (Washington, D.C)*, *10*(5), e02748. <https://doi.org/10.1002/ecs2.2748>

Watanabe, S. (2010). *Asymptotic Equivalence of Bayes Cross Validation and Widely Applicable Information Criterion in Singular Learning Theory*. <https://www.jmlr.org/papers/volume11/watanabe10a/watanabe10a.pdf>
